# Xbb subvariant of Omicron: Can the new wave sneak past your immune defense?

**DOI:** 10.1097/JS9.0000000000000042

**Published:** 2023-01-12

**Authors:** Mahmoud T. Hefnawy, Nour Shaheen, Omar A. Abdelwahabd, Mariam Tarek Desouki, Almoatazbellah Attalla, Abdurthman Khaity, Ala’ A. Rababah, Abdelrahman Mohamed, Youssef Soliman, Rehab A. Diab, Asmaa Elganady, Samar A. Amer

**Affiliations:** a Faculty of Medicine, Zagazig University, Egypt; b Department of Community Medicine and Public Health, Faculty of Medicine, Zagazig University, Zagazig; c Faculty of Medicine, Alexandria University, Alexandria; d Faculty of Medicine, Al-Azhar University; e Medical Research Group of Egypt (MRGE), Cairo; f Faculty of Medicine, Assiut University, Assiut, Egypt; g Department Internal Medicine, King Hussein Medical Center, Amman, Jordan; h Faculty of Medicine, Elrazi University, Khartoum, Sudan

Since the emergence of coronavirus disease-2019 (COVID-19), many subvariants have emerged from the mother Omicron variant, such as the BA.4, BA.5, and the recent BA.2.75^1^,^2^. Despite the reports of COVID-19 cases and deaths will have decreased globally by the end of 2022, there are new coronavirus omicron subvariants that are technically expected to release a new wave of infections across many parts of the world as the recent XBB, which is a new rapidly emerging omicron subvariant that has first been detected in August 2022 in India and Singapore then rapidly detected in 35 different countries with 1.3% global prevalence by October 2022 (Fig. 1). Despite its rapid transmission rate, the subvariant uses the same transmission methods as the omicron variant, which are droplet and contamination transmission, so experts believe that it is the most immune-evasive subvariant of omicron, with the highest risk of reinfection, according to World Health Organization (WHO) data^3^. In Singapore, XBB increased from 22 to 54% of regional cases in a single week, outpacing the dominating BA.5 and BA.2.75 strains from the previous week^4^; similarly, in Bangladesh, where XBB represented 85% of COVID cases within only weeks of initial detection, this indicates the unforeseen transmissibility of this subvariant^5^,^6^.

**Figure 1:**
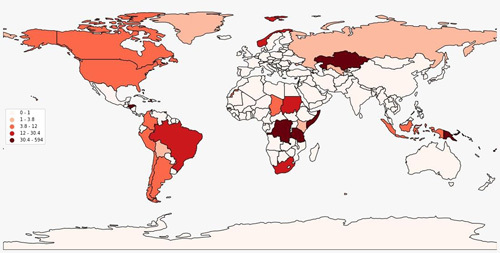
A map showing the geographical distribution of XBB subvariant according to total variant sequences as reported by GISAID.^3^

## The genome structure of XBB subvariant

**Figure 2:**
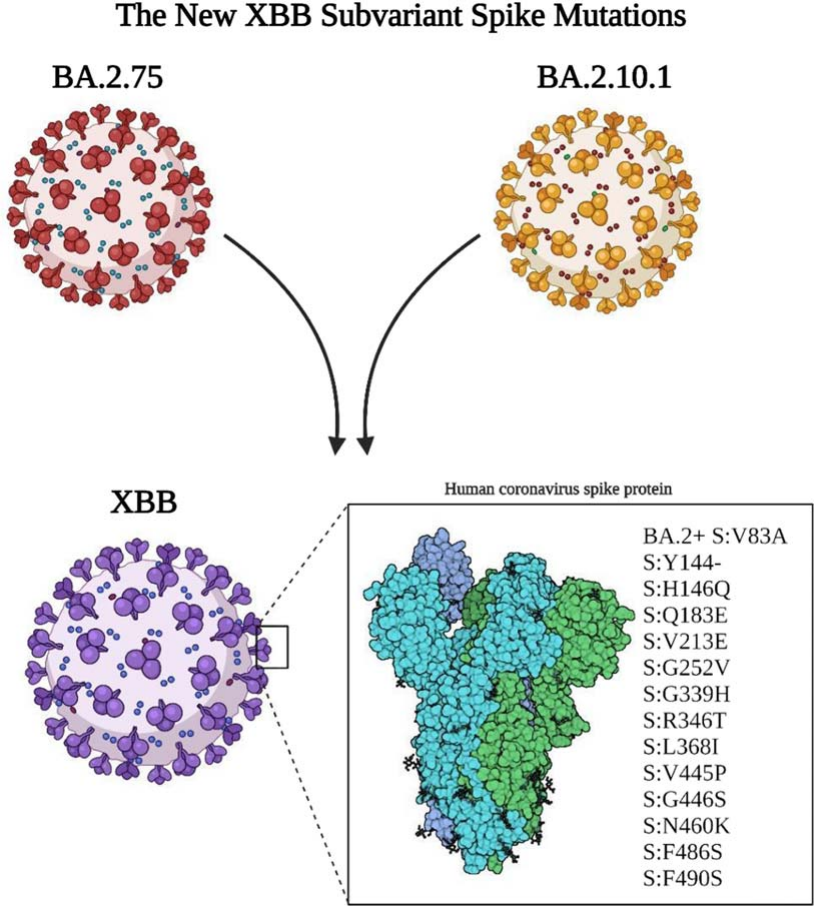
The spike mutations of the new XBB subvariant according to WHO.^7^

The XBB is a recombinant of BA.2.75 and BA.2.10.1 omicron subvariants in which the two subvariants infect the same cell allowing the exchange of genetic material of both, producing an entirely new hybrid subvariant, as identified by the recent updates of epidemiological week 40 from the Global Initiative on Sharing Avian Influenza Data (GISAID)^3^. XBB subvariant has at least seven new mutations from the original virus in the spike protein binding site. The recently detected spike mutations are BA.2+S: V83A, S:H146Q, S: Y144-, S: Q183E, S: G252V, S: V213E, S: G339H, S: L368I, S: R346T, S: V445P, S: N460K, S: G446S, S: F490S and S: F486S^7^. The XBB genome even showed multiple sublineage divisions as the XBB.1 and XBB.2, and XBB.3 as identified by the COVID-spectrum gene identifier, but these sublineages did not yet show a particularly warning spread as the original XBB 8,9 (Fig. 2).

**Figure 3:**
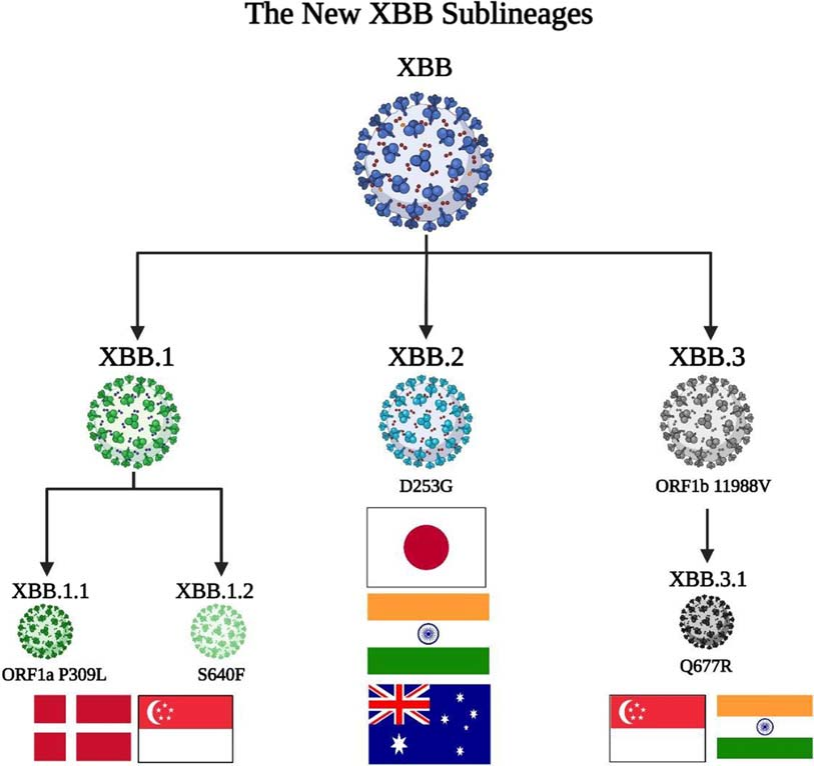
A brief of XBB sublineages.

One small mutation in the XBB spike protein generated the XBB.1 sublineage that was first discovered in the United States on September 15; GISAID statistics showed that it accounts for about 0.26% of cases that have undergone genetic sequencing in 15 days. Also, the data from GISAID revealed that XBB.1 has two subline divisions, XBB.1.1 with ORF1a P309L mutation and XBB.1.2 with an additional S640F spike mutation, and both can be found in Denmark and Singapore. The XBB.2 sublineage has a specific D253G spike mutation and could be found in Japan, India, and Australia. The XBB.3 sublineage has ORF1b 11988V mutation, and like the XBB.1, it has other division forms as the XBB 3.1 with Q677R spike mutation and was first detected in Singapore and India. This is the initial data about XBB spike mutations, as reported by experts so far^10^ (Fig. 3).

## The clinical picture of the XBB subvariant

Generally, the Omicron variants’ patients are commonly presented with prodromal symptoms, such as pyrexia, cough, and malaise. In addition, hypogeusia, anosmia, pharyngitis, skin rashes, and headaches also were reported^11^,^12^. BA.1 and BA.2 subvariants’ predominant forms reveal features like tiredness, fever, runny nose, and cough. At the same time, severe throat aches and general weakness were determined in patients with BA.4 and BA.5 subvariants^1^. Regarding the clinical diagnosis and available data of the XBB subvariant, most cases in India and Singapore demonstrated the same clinical consequences of previous Omicron sublineages but with discrepancies in some symptoms^3^. According to WHO, the probable effect of the XBB subvariant is profoundly impacted by the regional immune geography. However, these outcomes were gathered from sentinel countries and could not be completely pervasive in an alternative area. Accordingly, laboratory-based efforts are necessary to promptly create such assessments with global intelligibility^3^.

## Immune reactions and expected response to BA.5 vaccine

The current BA.5 vaccination boosters and herd immunity may not offer adequate coverage against infection with the new subvariants. In a study examining the neutralizing activity against six recently emerging Omicron subvariants (BQ.1.1, BF.7, BA.5, BA.2.75.2, BA.4.6, and XBB.1) using serum from people who received four doses of parental mRNA vaccination or a BA.5-bivalent booster, XBB.1 showed the highest amount of immune evasion, indicating the possibility of these new subvariants unseating the dominant BA.5 in circulation. However, the BA.5-bivalent booster produces superior neutralization against the recently developed XBB subvariant than the primary mRNA vaccination^13^,^14^.

There are higher neutralization reactions against the new omicron subvariants, including XBB, among people with a history of COVID-19 infection after vaccination with the BA.5-bivalent vaccine^14^. However, Reinfections mainly affected people who had contracted the disease before Omicron. There is currently no evidence in favor of escape from recent immunological reactions brought on by other Omicron lineages. Also, the regional immunological landscape, which is influenced by the magnitude and timing of prior Omicron waves as well as the COVID-19 vaccination coverage, appears to be a key factor in determining whether the increased immune escape of XBB is sufficient to drive new infection waves^3^.

Although the XBB variant developed its way of evading the immune system by acquiring mutations outside the spike RBD, robust immunity is developed by people formerly infected by BA.2 and BA.5 variants. Moreover, getting infected with the recent BQ.1 lineage offers double immunity, as it may somewhat protect XBB variants.^15^

However, the high transmissibility and immune evasion recorded of XBB subvariant that have the potential to establish a new worldwide wave of COVID-19 infection, there are no yet sufficient strong evidence data about the new subvariant spread, especially in the crude developing countries that lack both the vaccinations coverage and the surveillance of new COVID-19 infections. Further studies are recommended to report the neutralizing effect of the current vaccines with the new XBB subvariant.

## Provenance and peer review

Not commissioned, internally peer reviewed.

## Ethical approval

NA

## Sources of funding

NA

## Author’s contribution

All authors have contributed to writing and reviewing the manuscript.

## Conflicts of interest

All authors declare no conflict of interest.

## Guarantor

Nour Shaheen.
